# Operational Characteristics of Women Street Food Vendors in Rural South Africa

**DOI:** 10.3389/fpubh.2022.849059

**Published:** 2022-07-13

**Authors:** Tjale Cloupas Mahopo, Cebisa Noxolo Nesamvuni, Azwihangwisi Edward Nesamvuni, Melanie de Bryun, Johan van Niekerk, Ramya Ambikapathi

**Affiliations:** ^1^Centre for Sustainable Agriculture, Rural Development and Extension, University of the Free State, Bloemfontein, South Africa; ^2^Department of Nutrition, University of Venda, Thohoyandou, South Africa; ^3^Department of Public Health, Purdue University, West Lafayette, IN, United States

**Keywords:** operational characteristics, street food enterprises, street vendors, South Africa, women's employment

## Abstract

**Objective:**

To characterize the operations of the street food enterprise in the Vhembe district, focusing on business profile, sold foods, inputs, pricing, record-keeping practices and total running cost.

**Methods:**

A descriptive cross-sectional face-to-face study of 511 vendors was done using a structured researcher-administered questionnaire comprising demographic and operational characteristics. Convenience sampling was used to choose the vending sites. Chi-square tests were conducted between four categorical variables (gender, age, marital status and citizenship) and operational characteristics. *P*-values were considered significant at *p* < 0.05. However, a Bonferroni adjustment decreased the significant value to *p* < 0.013.

**Results:**

The findings highlight the dominance of single middle-aged (35–44) women (63.1%) with some high school education. About 14% migrated from Zimbabwe. Most vendors were owners (86.1%), with 70.0% in business for at least 1–10 years. Food sold included mielie pap (stiff porridge) served with beef or chicken, sometimes with vegetables. Plate prices were R40.00 (2.6 USD) for a full plate and R30.00 (2 USD) for half a plate. The typical street food consumers were government officials, middlemen, and schoolchildren. Social media such as Whatsapp were used to communicate between the street food vendors and customers. The results highlight poor managerial skills as only 15.5% kept records, most of which were sales records (59.5%). On average, street vendors made a monthly profit of R3200.00 (213 USD) while spending R1800.00 (120 USD) on daily running expenses. There were statistically significant variations in some operational characteristics of vendor variables and gender, age, marital status, and citizenship.

**Conclusions:**

There is a need for capital and management for small businesses and food training for rural street food vendors. Therefore, government officials, policymakers, and NGOs could target street vendors to offer training and microfinance to improve their business skills while promoting food safety and consumption of nutritious foods.

## Introduction

Foods sold on the street have become a common source of food consumption in many urban towns and cities. Street foods are foods prepared on the street and ready to eat at home or consumed on the street without further preparation (1). Street foods mainly consist of staple food served in various forms and combined with side dishes such as stews, gravies and snacks such as meat, fish, and cereal-based ready-to-eat foods (2, 3). Statistics South Africa and StatsSA (2020) reported that an estimated 18% of citizens to work in the informal sector as the main source of employment (4). Studies highlight an estimated one million street vendors in South Africa, of which over 70% sold food (5).

Street foods are less costly and an accessible means of obtaining a nutritionally balanced meal away from home for many low-income people (3, 6, 7). The street food sector in South Africa has been regarded as an essential channel for food access for a food-insecure population where it reflects a core aspect of low and middle-income countries, including South Africa's poor and vulnerable population (7). Even though its economic contribution goes overlooked on many occasions (8), street food enterprise is the single most significant employers in the informal sector due to its economic contribution (4, 9).

Informal enterprises' success depends on finance, the entrepreneur's characteristics, the business's location, and government support (10). Moreover, the government has established the Ministry of Small Business Development to develop Small and Medium Enterprises (SMEs). However, much support has been focused on the formal business economy and private franchises, leaving the informal sector, such as street food vending (11, 12). A previous study has reported that informal vending is not recognized because the economic activities that operate outside accepted norms of society are not regulated or registered with the government and are non-taxable (12). However, informal businesses in Gauteng are prioritized over other province (5).

The South African government recognizes the informal economy as a viable and essential form of employment and enabler of fiscal mobility for the poor (13). Studies in South Africa (6, 10, 14) and other countries (15, 16) indicated that women entrepreneurs engage in this sector for their entire working years because of a lack of opportunities in the formal sectors. Informal workers are overwhelmingly poor, dominated by young females (16), and have no support for their informal businesses to succeed (17). Moreover, the lack of formality due to poor governmental support contributes to the failure of the informal sector (18–20). This trend has also been documented in most African countries where street food vending is the leading source of income for the poor and vulnerable population, including women (16, 21, 22).

Studies conducted in the Limpopo Province (10, 14, 22) indicated that small-scale food vending contributes significantly to the livelihoods of the poor. Despite recognizing that street food vending forms most of the informal employment sector, the lack of formality means little is known of its operation. The lack of formality could be because no consultation of the street food vendors on any policies developed and implemented for managing their operation (23–25). Street food vending as an enterprise is often overlooked during policy formulation and disregarded in business strategy too easily (13). Nevertheless, these studies also indicate that regulations and by-laws governing street vending operations in South Africa are in place, although with a lack of enforcement by officials (25–29). Given the importance of street food in society, a better understanding of street food vendors in different settings is necessary to characterize its operation.

Limpopo province has the highest number of street food vendors, mainly concentrated in the Vhembe district. Hence the Vhembe district was selected for this study. Most street food vendors are found in public places such as taxi ranks and the road (22, 30, 31). The Vhembe district draws special attention in the province because of a high increase in cooked street foods (2, 14, 28, 31). This paper was the third phase of the main study, whose objective was to develop a model for street food vendors selling ready-to-eat foods in the Vhembe District. In phase one, Poter's competitiveness model investigates street food vendors' perception of vending competitiveness. Street food vendors perceived three main factors that determine the street food vending enterprise's competitiveness: chance, production, and government role factor conditions as determinants of enterprise competitiveness (Mahopo et al. forthcoming). In phase two, we reported that lack of opportunities and financial freedom as the main motivations for entry into the enterprise. We noted a need to establish local representative organizations with health authorities and enforce regulations by various stakeholders to assist street food vendors and their enterprises (32). Hence the current paper highlights the characterization of the street food vendors, focusing on business profile, employment, working hours, foods sold, inputs, pricing, record-keeping practices, total running cost and profit.

## Materials and Methods

### Study Design and Recruitment

The study was a cross-sectional survey that used well-structured close/open-ended questions and a convenience sampling technique to select 527 participating street food vendors during a transect walk-through. All available and willing street vendors selling cooked food were considered for the study. Due to the unavailability of the street food vendors' records, street food vendors were recruited in all towns. The consensus, therefore, deemed the sampling strategy to be used. However, only 511 street food vendors were considered for this study due to incomplete and missing information during data analysis. Data were collected on weekdays during busy times. Before the interviews, the study purpose, aims, and procedures were explained to the street food vendor. The street food vendors obtained informed, written consent, and confidentiality and anonymity were observed.

### Study Area

The study was conducted in the Vhembe district in the three towns: Makhado, Thohoyandou, and Musina. Vhembe district has an estimated population of 1.2 million (4). Most of the residents are women, and Thulamela has the highest population (females 55%) compared to Musina (52%) and Makhado (33). It covers an area of 21,402 square kilometers of mostly rural land. Due to the high level of unemployment (41.1%) (34), residents of the district have developed several survival strategies, including the street food enterprise at major trading points within the local municipalities. Thohoyandou and Makhado are big cities, relatively developed and economically vibrant cities (4). Musina remains relatively under-developed with a high incidence of shortage and constant migration of some of its active labor force to the North Vhembe district at the border of Zimbabwe.

### Inclusion and Exclusion

The researchers approached street food vendors selling cooked food. The sample frame included all street food vendors selling cooked foods in the Vhembe district. We excluded street food vendors younger than 18 years and those who provided incomplete information.

### Study Duration

The study period was 3 months, from July to September 2019.

### Sample Size Determination and Sampling

The municipalities had no records indicating the locations of street food vendors. Hence a non-probability sampling strategy was used as the research population is unknown (35). In the Vhembe district, there are four main towns, of which three of the most populated town were selected (Thohoyandou, Musina and Makhado). The estimated population size of these three towns was that Thohoyandou had 69,000 Musina 43,000, and Makhado population size 23,000. Convenience sampling was used to select study participants from all towns and three towns. The researcher established and maintained a complete list of the primary unit component from the Vhembe district's municipalities. All vendors encountered were approached subject to eligibility criteria.

### Data Collection Tools

An adapted questionnaire was used to collect data on street food vendors' demographic and operational characteristics (see study tools below). The study instrument was piloted among 20 selected street food vendors operating in Sibasa, approximately 6 kilometers outside Thohoyandou.

### Demographic Variables

Four demographic variables, gender, age, marital status, and citizenship, were used to characterize the operations of the food vendors.

### Operational Characteristics

The operational variables assessed were (1) business profile, (2) foods sold and sources of inputs, (3) food per plate pricing, (4) target market, (5) enterprise record-keeping practices, and lastly, (6) total cost and profit.

### Data Analysis

All analyses were carried out using the Statistical Package for Social Sciences (SPSS version 26). The median and IQR were calculated because the data were skewed for most variables. The test of four *a priori* hypotheses were assessed to characterize the operations of the street food vendors in terms of their background characteristics. Chi-square tests were conducted between four categorical variables (gender, age, marital status, and citizenship) and operational characteristics. *P*-values were considered significant at *p* < 0.05. However, a Bonferroni adjustment decreased the significant value to *p* < 0.013.

## Results

### Demographic Information

Table 1 reports the street food vendors' demographic information based on the town's location. Among the 511 street food vendors participating in the study, 90.2% were women, and 43.6% were single aged 35–44 years (43.6%). At the same time, those aged 45 years were 3.8 and 32.7% of males and females, respectively. A few young adults (4.1%) participated in the study. Thohoyandou (60.1%) had the most street food vendors, followed by Musina (32.9%) and Makhado (7.0%). Most of the street vendors were never married (43.6%), with just over a quarter of the participants married (28.0%), and a few were widowed (3.3%). Most of the single (26.0%) and married (16.0%) participants were from Thohoyandou. Regarding educational status, more than half (50.5%) of the participants had some high school education, while 28.8% obtained a matric certificate, and only a very few (3.7%) never attended formal education. Most street vendors were owners (86.1%) of their businesses and only 2.2% were employees. Less than 15% (14.0%) of the migrants were of Zimbabwean citizenship, with the majority (85.7%) of the street vendors from South Africa.

**Table 1:** Demographic information of street food vendors.

**Variables**	**Musina frequency (%)**	**Makhado frequency (%)**	**Thohoyandou frequency (%)**	**Total frequency (%)**
Sample size	168 (32.9%)	36 (7.0%)	307 (60.1%)	511 (100%)
**Gender**				
Male	9 (1.8%)	1 (0.2%)	40 (7.8%)	50 (9.8%)
Females	159 (31.1%)	35 (6.8%)	267 (52.3%)	461 (90.2%)
**Marital status**				
Single	83 (16.2%)	5 (1.0%)	135 (26.4%)	223 (43.6%)
Married	50 (9.8%)	9 (1.8%)	84 (16.4%)	143 (28.0%)
Living with partner	29 (5.7%)	20 (3.9%)	68 (13.3%)	117 (22.9%)
Divorced	1 (0.2%)	0 (0.0%)	10 (2.0%)	11 (2.2%)
Widowed	5 (1.0%)	2 (0.4%)	10 (2.0%)	17 (3.3%)
**Age categories**				
18–24 years	10 (2.0%)	1 (0.2%)	10 (2.0%)	21 (4.1%)
25–34 years	33 (6.5%)	2 (0.4%)	46 (9.0%)	81 (15.9%)
35–44 years	75 (14.7%)	23 (4.5%)	125 (24.5%)	223 (43.6%)
45–44 years	33 (6.5%)	9 (1.8%)	90 (17.6%)	132 (25.8%)
55–64 years	11 (2.2%)	1 (0.2%)	35 (6.8%)	47 (9.2%)
65–74 years	3 (0.6%)	0 (0.0%)	1 (0.2%)	4 (0.8%)
75 and above	3 (0.6%)	0 (0.0%)	0 (0.0%)	3 (0.6%)
**Educational status**				
No formal schooling	10 (2.0%)	1 (0.2%)	8 (1.6%)	19 (3.6%)
Primary education	26 (5.1%)	1 (0.2%)	30 (5.9%)	57 (11.2%)
Some high school	101 (19.8%)	19 (3.7%)	138 (27.0%)	258 (50.5%)
Matric	29 (5.7%)	14 (2.7%)	104 (20.4%)	147 (28.8%)
Diploma	2 (0.4%)	1 (0.2%)	23 (4.5%)	26 (5.1%)
Degree	0 (0.0%)	0 (0.0%)	4 (0.6%)	4 (0.6%)
**Ownership**				
Full owner	129 (25.2%)	25 (4.9%)	286 (56.0%)	440 (86.1%)
Co-owner	34 (6.7%)	8 (1.6%)	18 (3.5%)	60 (11.7%)
Employees	5 (1.0%)	3 (0.6%)	3 (0.6%)	11 (2.2%)
**Citizenship**				
South Africa	110 (21.5%)	22 (4.3%)	304 (59.5%)	436 (85.3%)
Zimbabwe	58 (11.4%)	14 (2.7%)	3 (0.6%)	75(14.7%)

### Business Profile of the Street Food Vendors

As shown in Table 2, 70.1% of the participants were in the business for 1–10 years, indicating that most street food enterprises are relatively well-established. Only 10.0% were still new in the street food business. The median weekly money made from the business was R1300.00 (86 USD). The monthly income from the street food vendor's household income was R4000.00 (266 USD). For an average street food vendor in need of financial assistance, most participants (65.0%) indicated getting money from their savings to start up, while very few indicated money lenders (1.0%), banks (0.8%), and others (1.4%) sources. These results highlight the lack of financial support from commercial banks and street food vendors. The results also show that street vendors save money physically in their pocket (97.1%) and a box (12.7%) to keep money safe while working. At the same time, very few used tills (1.6%). Only 5.1% of the vendors reported saving money in the banks (5.1%).

**Table 2:** Business profile of the street food vendors.

**Variable description**	**Frequency (%)**	**Median (IQR)**
**Age in the business**		
Less than a year	51(10.0%)	
1–10 years	360 (70.1%)	
11–20 years	79 (15.5%)	
21–30 years	18 (3.5%)	
More than 30 years	3 (0.9%)	
Average income from stall/week (*R*)		1,300 (1,900)
Average household income (*R*)		4,000 (4,900)
**Financial assistance**		
Bank loan	4 (0.8%)	
Moneylenders (loan sharks)	5 (1.0%)	
Savings	332 (65.0%)	
Other (not specified)	7 (1.4%)	
**Storage of money**		
In a till	8 (1.60%)	
In a box	65 (12.7%)	
Pocket	408 (97.1%)	
Bank	26 (5.10%)	
Other (unspecified)	1 (0.20%)	

### Employment and Operational Hours of the Street Food Vendors

Table 3 reports the street food vendors' employment and hours worked. The enterprise had at least one permanent person working. Although there was no difference between the number of days worked, the median hours worked by the unskilled was 11 h compared to 9 h of the skilled street food vendors on weekdays and Saturdays, respectively. There was no difference in days worked between the skilled and unskilled, as shown by a median of 300 days yearly. The median amount paid for unskilled labor was R1470.00 (98 USD), while skilled laborers were paid a median of R1400.00‘(93 USD) per month.

**Table 3:** Employment status and operational hours of street food vendors.

**Variables**	*Skilled (*N* = 33)	**Unskilled (*N* = 144)**
	**Median (IQR)**	**Median (IQR)**
Permanent employment status	1 (0)	1(0)
Total days worked	6 (1)	6 (1)
Weekday hours	9 (3)	11 (3)
Saturday	9 (3)	11 (2)
Sunday	0 (8)	0 (12)
Holidays	9 (3)	9 (5)
Days worked per year	300 (0)	300 (50)
^∧^Salary bill (R)	1,400.00 (0)	1,470.00 (975.00)

*Skilled, received some unofficial training; unskilled, never had any training.

*^∧^currency: 1USD = R15.00*.

### Food Sold and Sources of Inputs Profiling

Table 4 reports the foods and sources of input. Street food vendors predominantly cooked meat (98.8%) and Millie pap (stiff porridge) (97.1%), a South African staple food. Three quarters (75.3%) of the vendor's cooked vegetables and 34% were served as gravy and chakalaka (8.2%). The various meats cooked by the street vendor were chicken (88.8%), beef (68.3%), beef offal/*magulu* (14.3%), and beef sausage (5.5%), with very few street vendors selling fish (1.4%) or pork (2.2%). The median cost of meat was R400.00 (27 USD). The street food vendors cooked and sold two main starchy foods, mielie pap (97.1%) and rice (14.1%), at R70.00 (5 USD) daily. Although vegetables formed part of the plate, only 60.1% sold green vegetables as part of the menu, with others serving gravy (34.8%) and chakalaka (8.2%) as supplements. The median cost of vegetables used for cooking was R 40.00 (3 USD). Food was mainly cooked on-site (93.3%), with owners (74.5%) as the prominent people cooking food and very few assisted by the employees (23.9%) or spouses (1.6%). Most street food vendors (86.3%) took left-overs home to eat with family members, while 0.2% reported selling leftovers.

**Table 4:** Inputs and inputs supplier profiling.

**Variable description**	**Frequency (%)**	**Median (IQR)**
**Foods mostly cooked (ready-to-eat foods)**		
Meat (R)		400.00 (240.00)
Chicken	454 (88.8%)	
Beef/ox	349 (68.3%)	
Wors	28 (5.5%)	
Pork	11 (2.2%)	
Beef Offal	73 (14.3%)	
Fish	7 (1.4%)	
Starch		70.00 (60.00)
Pap (R)	496 (97.1%)	
Rice	7 (14.0%)	
Vegetables (*R*)		40.00 (50.00)
Beans	9 (1.8%)	
Green leafy vegetables	307 (60.1%)	
Carrots	1 (0.2%)	
Gravy	178 (34.8%)	
Chakalaka	42 (8.2%)	
Cooking place		
At the site	477 (93.3%)	
Home	38 (7.4%)	
Person responsible for cooking		
Spouses	8 (1.6%)	
Employee	122 (23.9%)	
Self	381 (74.5%)	
Preservation of leftovers		
Take home to eat	441 (86.3%)	
Sell next day	1 (0.2%)	
Sell at a lesser price	25 (4.9%)	
Throw it away	22 (4.3%)	

### Street Food Plate Pricing

Table 5 reports street food plate pricing. The median price for a full plate of chicken was R40.00 (2.7 USD), with a minimum of R20.00 (1.3 USD) and a maximum of R140.00 (9.3 USD). The median price of a full plate of beef was R40.00 (2.7 USD), with a minimum of R25.00 (1.7 USD) and a maximum of R75.00 (5 USD). Moreover, a median price of a half-plate was R30.00 (2 USD).

**Table 5:** Street food plate pricing.

**Variable items**	**Median (IQR)**	**Min**	**Max**
**Plate prices**			
Beef/pork full plate	R40.00 (R10.00)	R25.00	R76.00
Beef/pork half plate	R30.00 (R5.00)	R15.00	R55.00
Chicken full plate	R40.00 (R10.00)	R20.00	R140.00
Chicken half plate	R30.00 (R10.00)	R15.00	R70.00

*^a^Full plate consists of 4 or more servings of meat*.

*^b^Half a plate consists of 2–3 servings of meat*;

^*^Currency: 1USD = R15.00.

### Target Market

Table 6 reports the target markets of the street food vendors. Most participants reported rural consumers (81.2%) as their primary customers, while 52.3 and 58.7% reported middlemen and government officials. Moreover, 40.9% reported school children as their target market. Most participants (72.0%) highlighted using personal visits/face-to-face, whereas 62.4% used phone calls/WhatsApp to retain customers. Some street vendors indicated that word of mouth (2.7%) and posters (0.6%) communicate with their customers about their services and food sold.

**Table 6:** Street food vending target market.

**Variables**	Frequency*(%)
**Target market**	
Rural customers	415 (82.2%)
School children	209 (41.5%)
Government employees	300 (59.5%)
Middlemen	267 (53.0%)
Other	9 (1.8%)
**Mode of communication**	
Personal visits	368 (72.0%)
Phone call/WhatsApp	319 (62.4%)
Recruit professionally	22 (4.3%)
Word of mouth	14 (2.7%)
Posters	3 (0.6%)
Nothing	7 (1.5%)

^*^Number of responses.

### Record Keeping

Table 7 highlights that all street food vendors keep similar records. However, some participants (15.5%) could only keep business records. Most records were sales (59.5%), and 46.8% could keep production records. More than a quarter of the participants (36.7%) kept marketing records. Most of the street vendors indicated that due to lack of infrastructure, they sometimes keep stock (69.9%) in their storeroom (30.3%), homes (27.2%) and stalls (21.0%). Some street vendors reported using rented rooms (19.6%) in the vicinity to keep their stock safe. The practice of stock inventory was not typical because only 11.2% of street vendors could do so.

**Table 7:** Street food vendor's record-keeping practices.

**Variables**	Frequency*(%)
**Keep records**	
Yes	79 (15.5%)
No	432 (85.6%)
Production	37 (46.8%)
Marketing	29 (36.7%)
Sales	47 (59.5%)
Yes	357 (69.9%)
No	154 (30.1%)
Home	97 (27.4%)
Stall	75 (21.2%)
Storeroom	108 (30.5%)
Rented room	70 (19.8%)
Other	4 (1.1%)
Yes	54 (11.2%)
No	427 (88.8%)

^*^Number of responses.

### Total Running Cost and Profit

Table 8 shows that the daily median total running cost was R1800.00 (120 USD). Monthly street food vendors could make a median monthly profit of R3200.00 (213 USD). Only 32% had employees with a monthly expenditure of R1400.00 (93 USD). Street food vendors spent R430.00 (29 USD) and R340.00 (23 USD) on gas and transportation. Other business expenditures were electricity (R100.00) (7 USD), water (R100.00) (7 USD), cell phone (R100.00) (7 USD) and bank charges (R50.00) (3 USD).

**Table 8:** Total running cost and profit.

**Variable description**	**Frequency (%)**	**Median (R)**	**IQR (R)**
Total running cost/day	494 (97.0%)	1,800.00	2,200.00
Total profit	473 (93.0%)	3,200.00	3,900.00
**Monthly expenditure**			
Electricity	191 (37.4%)	200.00	150.00
Gas	402 (79.0%)	430.00	241.00
Employees	162 (32.0%)	1,400.00	1,000.00
Transportation	339 (66.3%)	340.00	250.00
Water	380 (74.4%)	100.00	100.00
Cell phones	329 (64.4%)	100.00	70.00
Bank charges	21 (4.1%)	50.00	86.00

*Rand (R), Currency; IQR, Interquartile range*.

### Association Between Operational Characteristics With the Four Demographic Characteristics of the Street Food Vendors

Table 9 reports the association between the operational variables with the four demographic characteristics of the street food vendors. The test of four a priori hypothesis was assessed using four independent categories. Chi-square associations were determined with a Bonferroni adjusted alpha level of 0.013. The street vendors in the four categories differed in gender, age, marital status, and citizenship level. There was no significant difference in gender, age, or marital status regarding the days worked. However, there was a statistical difference in the number of days worked and nationality. South Africans (76.6%) worked 6–7 days a week compared to 94.7% of Zimbabweans (*p* < 0.001).

**Table 9:** Operational variables for which there was a significant difference in the characteristics of the respondents using Bonferroni adjusted level.

**Variables**	**Gender**	**Age**	**Marital status**	**Citizenship**
**Operational working day**				
Total hours worked on a weekday				<0.001
Total days worked				<0.001
Total days on a sunday		<0.001		<0.001
Total days worked sunday				<0.001
**Types of food sold**				
Meat	a	a	a	a
Pap	a	a	a	a
Vegetables	a	a	a	a
**Sell same food always**				<0.001
**The site where food is sold**				
**Preservation of leftovers**			<0.001	
**Ownership**				<0.001
**Number of employees**		0.012		
**Inputs cost**				
The average cost of meat			0.004	
The average cost of millie pap		<0.001	0.002	0.003
Total running cost			0.004	
Total profit	0.008			0.008
**Expenditure**				
Amount spent on employees		0.004		<0.001
Transportation		0.003	<0.001	<0.001
Cell phones		<0.001		0.001
Water			<0.001	<0.001
**Plate pricing**				
Chicken full	<0.001			
Chicken half	<0.001			<0.001

*a-refers to the same food type that are sold throughout the year*.

Total hours worked on a Sunday were not statistically different based on gender or marital status. However, there was a statistical difference in the hours worked on a Sunday based on age, with 50.0% of 65–74-year-olds working more than 8 h on a Sunday compared to lower percentages in the younger age groups (*p* < 0.001). Similarly, there was a statistical difference in hours worked on a Sunday based on citizenship, with 6.4% of South Africans working more than 8 h on a Sunday compared to 45.3% of Zimbabweans (*p* < 0.001). Moreover, a significant difference was observed between marital status and the preservation of leftovers, with the separated (100%), married (92.3%), and single (88.3%). Street food vendors take food for home consumption while 4.2 and 36% of married and single throw food.

Ownership had no statistical difference with age, gender, or marital status. However, a significant difference was observed between citizenship and full-ownership (*p* < 0.001), with 91.5% of South Africans as full-owners compared to 54.7% of Zimbabweans who own the businesses. Similarly, a statistical difference in age and number of employees had statistical significance (*p* = 0.012), as more than 50% of the vendors aged 65 years and above have at least 1–3 employees.

There was no significant difference between gender, age, or citizenship status and the cost of meat. However, a significant difference was observed between marital status and the cost of meat, with single (6.7%) vendors who could purchase meat for more than R1,000 compared to married (3.5%), living with partners (1.7%) and the separated (9.1%). Most participants could afford to buy meat at a price ranging from R100–R500. A significant difference was also observed between the cost of mielie pap and age (*p* < 0.001). Compared to all marital statuses, vendors living with their partners (70.1%) could afford mielie pap ranging from R51 to R100 (*p* = 0.002).

Citizenship and selling price of mielie pap (stiff porridge) differed with most Zimbabweans (73.7%) selling at R51–R100 of mielie pap than 54.6% of South African. There was also a significant difference between total running costs with marital status (*p* < 0.004), where single (10.8%) as compared to married (5.6%) and living with partners (8.5%) could spend more than R5,000 on the total cost for the business. Furthermore, significant differences were observed regarding profit made in gender (*p* = 0.008) and citizenship (*p* = 0.008). Males (42%) in the study profited more than females (23.2%) and could make a monthly profit of more than R5,000. Moreover, only South Africans (26.4%) could make enough profit compared to Zimbabweans (17.3%).

Expenditure had differences as shown by the *p*-value of electricity (*p* = 0.004), the amount spent on employees (*p* < 0.001), transportation (*p* < 0.001), cell phone use (*p* < 0.001), and water (*p* < 0.001). Most of the time, South African street food vendors (11.9%) were the most to price the plate at more than R50 compared to the non-South Africans. The difference was only observed with half chicken plate pricing on meal pricing (*p* < 0.001).

## Discussion

This survey aimed to characterize the operations of the street food vendors in the Vhembe district. Previous street food vending studies have not characterized street enterprise availability and distribution across the Vhembe district towns. The predominance of women aged 34–44 in the present study is not surprising, although men were available. Lower than the monthly earnings of the informal sector employees reported nationally in 2016 (36). The reason for women dominating the street food vending in the study could be that the business is focused only on cooked meals where women are commonly involved in preparing and serving food. In South Africa, studies have reported women's dominance in street food enterprise (19, 31, 37) who depend on the business for social relief (4, 20, 38). A lack of employable skills, insufficient work experience, and inadequate financial resources are among the factors contributing to women's dominance in the sectors (28). Similar trends were observed in other African countries (15, 18, 20).

Food vending activities enhance income for some households to meet their additional financial needs (39). In the current study, most street food vendors were never married, which may be attributed to the declining marriage in South Africa amongst youth (40). However, in Uganda, single street food vendors engage in street food vending as their only source of income (41). It is documented that marital status is one reason for establishing the household's livelihood strategies for the consumers and vendors (42). Even though the current study highlights that the proportion of married street vendors was significant, married street food vendors could be engaging in the business as an additional financial resource (41).

Education is essential for the design of appropriate training interventions. In South Africa, studies have shown that most street food vendors are educated, with most having achieved secondary education or post-secondary/tertiary qualification (43). The Vhembe district reported a decrease of 1.8% in people without schooling (33). The current study also shows that most street food vendors are educated, contrary to other studies in part of the African countries (15, 16, 21). A highlight is that if vendors are trained adequately in business establishments, their business skills could improve while promoting food safety and consumption of nutritious foods. Even though it is documented in South Africa that educated vendors venture into the business because of a lack of employment opportunities in the formal market (44–47). Hence the need to acknowledge that many people engage in street food vending to survive and meet their basic needs due to poverty and unemployment and that it is not by choice, but the country's employment status forces them (48).

The vendors' business profile highlights that most worked long hours with the sole employer. Significant differences were observed between operational working hours and citizenship, where foreign nationals worked fewer hours than South Africans. According to the Basic Employment Conditions Act of 1997, an employee who works more than 5 days a week should work 8 h daily (49). Nevertheless, street food vendors worked more hours and days, contrary to the Basic Employment Conditions Act of 1997. Similar findings were also observed in Cape Town, where street food vendors worked more than the average population (2, 23). Although business hours tend to vary per enterprise, meal types are prepared and served because enterprise locations are influenced primarily by the specific days of the week, and it was not the case in the current study as the same foods were sold (50). Furthermore, males worked for longer hours on weekdays and weekends, especially Sundays, which could be attributed to female street food vendors having other responsibilities after work, such as taking care of families.

Similar to other studies (9, 20, 27, 28, 31, 51), this study showed that street food vending could reduce unemployment as 9.0% of the enterprises had employees. However, challenges such as lack of access to capital, with only 0.8% of the street food vendor who had access to financial assistance, could make it difficult for street food enterprises to strive (52). Street food enterprises' survival was based on vendors' savings and, at times, getting some financial assistance from the “loan sharks,” where they are expected to return the money with a certain percentage that could hinder profit. The current study's findings are not different from those of other studies (20, 53) that street food vendors source initial money from their savings to establish and sustain the enterprise. Furthermore, street food vendors borrow money from money lenders (loan sharks) for continued food business operation, although it comes at a cost (54).

Further attributes that need to be considered are expenditure and business record-keeping practices. A study in Ghana highlighted a need for policy interventions to improve the street food sector regarding managerial constraints (55). As reported in other studies (20), the current study highlighted that vendors spend more money on business expenses, including buying inputs, with little monthly profit. These results are consistent with a study in Ghana (55) that was a constraint to the growth of micro and small scale enterprises where increased inputs and business expenses were identified as a constraint to street food enterprise growth.

Street food is consumed mainly by working-class people who use public transport (3, 6, 19, 31). In Mexico, it is reported that those who commute long distances or lack time to prepare food at home consume most food outside their homes (56). This street food vendor location was also observed in other parts of the country (5, 10, 29) and Africa, although their location is sometimes illegal (57–60). Plate costs in the study remained affordable and attractive to the poor groups compared to the expensive meal of R70–R300 (5–20 USD) from a restaurant (https://www.numbeo.com/cost-of-living/in/Polokwane). With the association's help, street food vendors determined the standard price needed in the same vicinity. There is a need to strengthen this partnership amongst the street food vendors with the formal institution and train them in business management. Training will help street food vendors produce and circulate goods and services within the informal food vending, expanding job opportunities in the absence of formal employment.

Food sold in the current study included traditionally cooked starches such as pap and rice served with chicken/beef/pork, and very few vendors served fish. However, green leafy vegetables were uncommon. These foods were not different from food reported in other studies selling cooked street food (3, 23, 61) and highlight that if healthy food items are made available and affordable, people are more likely to consume them (56, 62). The limitation observed was that cooked food was not individually identified, and due to the nature of the study, portion sizes were also not being calculated. Other research (3) in the country highlighted the nutritional contribution of street food, citing its importance in providing energy and micronutrients to supplement the home meal. Street food vendors should be encouraged on the types of foods sold to customers to improve the nutritional quality of foods sold on the street.

Although there was no significant difference between gender and place food is cooked, the use of leftovers in the current study highlighted across all marital status that street food vendors take along their leftovers to use at home. All street food vendors could be encouraged to save on food and use food profitably rather than throwing and reselling, posing health issues. Like our findings, consuming leftovers is common among street food vendors (17). Although 56% of street food vendors indicated using the leftovers for the next day in Zimbabwe, posing health risks to customers (15), many (44%) still use the leftovers for home consumption. This indicates that the street food vending enterprise contributes to the food vendor's mass and family. In addition, studies conducted in South Africa highlighted that traditionally cooked street food microbial safety was surprisingly better than expected (9, 23). It is reported that the less availability of the microbial concerns in the cooked food could be because street vendors should ensure that the environment they are operating from is always kept clean (60). Such practices must be encouraged to all vendors selling cooked foods to avoid loss in the business and contribute to the family diet. Therefore, public health professionals should better engage the street food vendors by selling cooked food and keeping them safe for consumption to contribute to the household's nutritional wellbeing.

## Conclusions

The majority of our sample were women, who were single with some school education. The study also highlighted the presence of migrants working as street food vendors in the Vhembe district. Most vendors owned the business and have been operating for 1–10 years. Males comprised 10% of our sample, and indicated higher working hours than females. Food sold in the study were commonly consumed foods such as mielie pap, beef, chicken, and seasonal vegetables. The street vendors encountered many constraints, such as a lack of essential services, including water, financial assistance from the formal institution, energy, and transportation.

Moreover, street vendors portray poor managerial skills. However, street vending has shown that street food enterprise contributes to the local economy by offering employment. There is an urgent need for an intensive capital provision for street food vendors' businesses and a management food training that will equip these entrepreneurs, mainly in the grassroots communities of the Vhembe district of South Africa. Therefore, government officials, policymakers, and NGOs could target street vendors to offer training and microfinance to improve their business skills while promoting food safety and consumption of nutritious foods.

## Study Strength and Limitations

This is the first study to evaluate women's street food vendor business operational characteristics on a larger scale in the Vhembe district, Limpopo province. The study's findings can help customize supporting policies for different street food vendors to improve the country's economic status, informal business equity, food safety and nutrition security. Furthermore, our measurement tools were piloted, resulting in their increased reliability. However, this study had some weaknesses. First, the cross-sectional nature of the study design prevented us from making any causal conclusions between variables. Second, some explanatory variables relied on participants' self-reported data, prone to recall and social desirability biases. To minimize this bias, study participants were allowed adequate time to recall previous information as best as possible.

## Data Availability Statement

The data sets used or analysed during the current study are available upon reasonable request from the corresponding author.

## Ethics Statement

Ethical approval was obtained from the University of Venda (project No: SHS/19/NUT03) and the University of the Free State (project No UFS-HSD2019/0059). The researcher obtained approval from the Vhembe district municipalities and the street food committee to conduct the study. The researchers surveyed street food enterprises, interviewing street food vendors available and knowledgeable about the business. The business owner/employees were informed of the research objectives and conducted interviews to secure consent. The participants were informed of the purpose of the investigation before participating in the study. Furthermore, participants were given assurance of privacy, confidentiality, and anonymity regarding the information provided. Street vendors signed the consent form after explaining the study's aim and objectives.

## Author Contributions

TM, CN, and AN contributed to the conception and design of the study. JN, MB, and RA contributed to the manuscript writing. All authors reviewed the manuscript and approved the submitted version.

## Funding

This project was implemented with a grant from the Water Research Commission, South Africa [Project No: K5/2818/4]. Also, the University of Free State awarded a scholarship to a Doctoral student TM [Project Number: UFS-HSD2019/ 0059]. The University of Venda Capacity Development Program supported the Doctoral Student with Research Funds [Project No: SHS/19/NUT03].

## Conflict of Interest

The authors declare that the research was conducted in the absence of any commercial or financial relationships that could be construed as a potential conflict of interest.

## Publisher's Note

All claims expressed in this article are solely those of the authors and do not necessarily represent those of their affiliated organizations, or those of the publisher, the editors and the reviewers. Any product that may be evaluated in this article, or claim that may be made by its manufacturer, is not guaranteed or endorsed by the publisher.
